# Apoptosis deregulation in myeloproliferative neoplasms

**DOI:** 10.1590/S1679-45082013000400025

**Published:** 2013

**Authors:** Raquel Tognon, Natália de Souza Nunes, Fabíola Attié de Castro

**Affiliations:** 1 Faculdade de Ciências Farmacêuticas de Ribeirão Preto, Universidade de São Paulo, Ribeirão Preto, SP, Brazil

**Keywords:** Myelodysplastic-myeloproliferative diseases, Apoptosis/physiopathology, Proto-oncogene proteins c-bcl-2, Receptors, death domain

## Abstract

Philadelphia-chromosome negative chronic myeloproliferative neoplasms are clonal hematologic diseases characterized by hematopoietic progenitor independence from or hypersensitivity to cytokines. The cellular and molecular mechanisms involved in the pathophysiology of myeloproliferative neoplasms have not yet been fully clarified. Pathophysiologic findings relevant for myeloproliferative neoplasms are associated with genetic alterations, such as, somatic mutation in the gene that codifies JAK-2 (JAK V617F). Deregulation of the process of programmed cellular death, called apoptosis, seems to participate in the pathogenesis of these disorders. It is known that expression deregulation of pro- and anti-apoptotic genes promotes cell resistance to apoptosis, culminating with the accumulation of myeloid cells and establishing neoplasms. This review will focus on the alterations in apoptosis regulation in myeloproliferative neoplasms, and the importance of a better understanding of this mechanism for the development of new therapies for these diseases.

## RESUMO

As neoplasias mieloproliferativas crônicas cromossomo Filadélfia negativas são doenças hematológicas clonais que se caracterizam pela independência ou pela hipersensibilidade dos progenitores hematopoiéticos às citocinas. Os mecanismos celulares e moleculares envolvidos na fisiopatologia das neoplasias mieloproliferativas crônicas ainda não estão totalmente esclarecidos. Achados fisiopatológicos relevantes para as neoplasias mieloproliferativas crônicas estão associados às alterações genéticas como, por exemplo, a mutação somática no gene que codifica o JAK2 (JAK2V617F). A desregulação do processo de morte celular programada, denominada apoptose, parece participar da patogênese dessas desordens. Sabe-se que a desregulação da expressão dos genes pró- e antiapoptóticos promove a resistência das células à apoptose, culminando com o acúmulo das células mieloides e estabelecendo a neoplasia. Esta revisão enfocou as alterações na regulação da apoptose em neoplasias mieloproliferativas crônicas e a importância da melhor compreensão desse mecanismo para o desenvolvimento de novas terapias para essas doenças.

## INTRODUCTION

Apoptosis is a type of genetically regulated programmed cell death that is triggered when the cell is exposed to certain physiological, pathogenic, or cytotoxic stimuli. Cells in apoptosis display typical morphological characteristics, such as cell volume reduction, folds in the cytoplasmic membrane, condensation and fragmentation of DNA, maintenance of the integrity of organelles, with the formation of apoptotic bodies and posteriorly phagocytosis by macrophages^(1)^. This process is highly regulated by cell signaling routes and pro-and anti-apoptotic proteins that control the steps involved.

Several studies in the literature show that apoptosis deregulation is involved in the pathophysiology of numerous nosologic entities, such as degenerative and autoimmune diseases and neoplasms^(2,3)^. Alterations of the anti-apoptotic *BCL*-2 molecule were associated to prognosis and response to therapy in chronic lymphocytic leukemia, non-Hodgkin lymphoma, and acute myeloid leukemia^(4,5)^. Cells of patients with chronic myeloid leukemia (CML) present with resistance to apoptosis and alteration of *BCL*-2 family gene expression, such as *MCL*-1 and *BCL*-w, among others^(6)^.

This review focused on apoptosis mechanisms and collected data in the literature that deal with deregulation of the programmed cell death process in chronic Philadelphia (Ph) chromosome-negative myeloproliferative neoplasms (MPNs), such as polycythemia vera (PV), essential thrombocythemia (ET), and primary myelofibrosis (MF). Its objective was also to discuss the importance of a better understanding of this mechanism for the development of new treatments for these diseases.

### Philadelphia chromosome-negative chronic myeloproliferative neoplasms

According to the World Health Organization (WHO), PV, ET, and MF are classified as chronic Ph chromosome-negative (Ph-) MPNs^(7)^. These neoplasms result from clonal expansion of altered hematopoietic stem cells and are characterized by hematopoietic progenitor cell independence from or hypersensitivity to cytokines, bone marrow hypercellularity^(8)^, and by the possible presence of acquired mutations in genes JAK2, TET2, CBL, AXSL1, and MPL^(9)^. Diagnosis may be suspected after onset of initial symptoms, such as increased erythrocyte mass, leukocytosis, thrombocytosis, or various degrees of cytopenia. MPNs progression is characterized by the appearance of splenomegaly, medullary fibrosis, and/or transformation into the myelodysplastic syndrome or acute leukemia^(8,10)^.

Among the molecular alterations involved in the pathophysiology of MPNs, the description of the JAK2 tyrosine kinase gene mutation, in 2005, had a great impact. The mutation that occurs in JAK2 is isolated, and consists of the substitution in the G nucleotide base by T in the 1,849 position of the gene, which leads to the exchange of valine for phenylalanine in the pseudokinase JH2 domain of the polypeptide sequence of JAK-2. This tyrosine kinase is responsible for changes in signaling of the JAK-STAT route, which transduces the signal of cytokine receptors and growth factors vital for the development of myeloid lineage cells, such as the colony-stimulating factor (CSF) and thrombopoietin (TPO), among others. With the alteration, tyrosine becomes constitutively activated, increases signaling of the JAK-STAT route, activates the PI3K, RAS, and STAT5 signaling routes, and causes hyperresponsiveness to signaling of cytokines, increased cell proliferation, and resistance to apoptosis^(11,12)^.

In an attempt to elucidate how a mutation leads to the three disorders, PV, ET, and MF, several hypotheses have been raised: the presence of additional mutations; the occurrence of mutation at different stages of differentiation, differences in the percentage of mutated alleles and in genes of patients^(13)^. Other mutations, including in another JAK2 exon, have been reported in JAK2V617F-negative patients. Mutations in exon 12 of JAK2 also affect aminoacids located in the pseudokinase domain, suggesting a mechanism of action similar to that of JAK2V617F^(9)^.

Modifications in the regulation of the programmed cellular death process, named apoptosis, seem to contribute to the myeloaccumulation and myeloproliferation in MPNs^(14–16)^.

### Apoptosis and Ph-negative chronic myeloproliferative neoplasms

There are two primary signaling routes responsible for apoptosis activation: the intrinsic and the extrinsic routes. The intrinsic or mitochondrial route may be initiated by various apoptogenic stimuli, such as chemotherapy agents that cause DNA damage, rupture of microtubules, and deficiency or absence of cell growth factors. These stimuli trigger the release of apoptogenic factors of the inner space of mitochondria, such as cytochrome c, second mitochondria-derived activator of caspase/direct inhibitor of apoptosis protein-binding protein with low PI (Smac/DIABLO), Omi/Htr 2, apoptosis-inducing factor (AIF), and endonuclease G. Released cytochrome c binds to Apaf-1 (apoptosis protease-activator factor 1) and to procaspase-9, forming the complex called apoptosome. Caspase-9 is activated, promoting activation of caspase-3 and of the proteolytic cascade. The Smac/DIABLO factors neutralized the effect of proteins that inhibit apoptosis (IAPs), such as c-IAPs and Survivin, and amplify the apoptotic signal. It is believed that AIF and endonuclease G contribute to nuclear modifications, such as chromatin condensation^(17,18)^.

The extrinsic route is activated by death receptors of the tumor necrosis factor (TNF) family, among them, TNFR1 and TNFR2 (TNF receptors), CD95 (Apo-1/FAS), and *TRAIL R1/DR4* and *TRAIL R2/DR5 (*TNF *related apopstosis -inducible ligand-death receptors)*. When stimulated by their agonists (*e.g.*, FasL and TRAIL), they lead to trimerization of the receptor and recruitment of death domain (DD) adapting proteins (homotypic interaction) and the death effector domain (DED). This bond recruits the initiating caspases, such as caspase-8, and promotes the formation of the multimolecular structure called death-inducing signaling complex (DISC). Oligomerization of caspase-8 during the formation of DISC induces its activation by auto-cleavage and then the activation of executioner caspases, such as caspase-3, -6, and 7. The prototype of activation of the extrinsic route of apoptosis may be represented by stimulation of the Fas-FasL route^(17,18)^. Another set of ligands and receptors that are important for activation of apoptosis by death receptors are those already mentioned, TRAIL, a member of the superfamily of TNF, and its receptors DR4 and DR5. The executioner caspases act in various substrates in the cytoplasm and nucleus, resulting in cellular death by apoptosis. The extrinsic route is regulated primarily by the caspase-8-like (FLICE)-inhibitory proteins (*C-FLIP*) molecules that possesses a domain very similar to that of caspase-8, but with no catalytic activity, and by the *FAS* apoptosis inhibitory molecule (FAIM) that antagonizes the bonding of FAS to its receptor and interferes in the expression of C-FLIP^(19)^. Activation of the intrinsic route is controlled primarily by the proteins from the *BCL-2* family, whose members are the anti-apoptotic proteins (BCL-2, BCL-X_L_, BCL-W, MCL-1, and A1), and pro-apoptotic proteins (Bax, Bak, Bad, Bid, Bim, Bok, Bik, BMF, Boo, BCL-X_S_, PUMA, and NOXA)^(18,19)^.

Besides the regulatory molecules of apoptosis that belong to the death receptor route and of members of the *BCL-2* family, we highlight the IAP that impede the executioner caspases. This family of proteins is composed of numerous members, and among them, the most often studied are XIAP, CIAP-1, CIAP-2, and SURVIVIN. We point out as functions of the IAPs, the inhibition of effector caspases 3 and 7, and the activation of caspase 9^(2)^.

In 1998, Fernandez-Luna et al. published a literature review and included data from their own laboratory about the pathogenesis of PV. They concluded that, in PV, other factors besides erythropoietin (Epo) would play an important role in the production of erythroid cells, among them, IGF-1, which besides other effects, could interfere in apoptosis by increasing the expression of *BCL-X_L_*, elevated in PV, causing the accumulation of cells in the absence of erythropoietin^(20)^. In 2006, Zeuner et al. analyzed the differentiation of erythroid cells in cell cultures and observed that the patient's cells that were PV JAK2 V617F-positive gave origin to a greater number of cells in the presence of inhibitors of the extrinsic route of apoptosis, showing lower activation of caspase induced by stimulation of this same route, and yet, that these patients presented with greater expression of the anti-apoptotic *C-FLIP* short^(21)^.

In 2004, Zhang et al. evaluated the expression of *BCL-X_L_* during megakaryocyte differentiation in patients with ET, and noted that the expression of this protein diminished early in *in vitro* megakaryocyte cell cultures, in the presence of TPO. This damage would suggest that the deregulation of this expression could explain, at least in part, the super production and differentiation of platelets, since *BCL-X_L_* is essential for megakaryocytopoiesis^(22)^. Florena et al. showed in biopsies of patients with ET and MF, the increased marking of BCL-X_L_ in ET, and in MF, elevated marking for Bax, Bad, and caspase-3 in megakaryocytes^(23)^.

**Figure 1:**
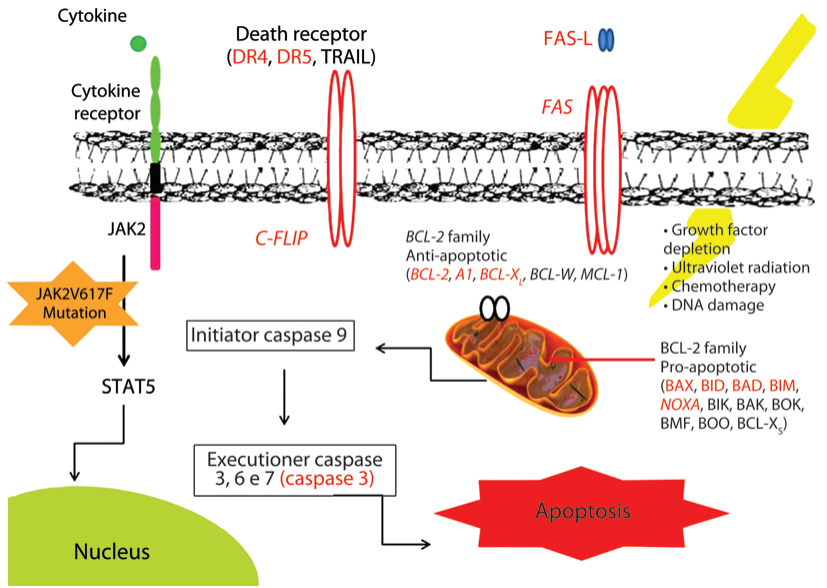
Representation of the extrinsic and intrinsic routes of cellular apoptosis and of the JAK-STAT route in cells of patients with myeloproliferative neoplasms (polycythemia vera, essential thrombocythemia, and primary myelofibrosis). The molecules that regulate apoptosis, with altered expression in these neoplasms, are highlighted in red. Illustration: Natália de Souza Nunes

Gasparotto et al. detected in CD34^+^ cells and leukocytes of patients with PV, alteration of the expression of anti-apoptotic genes *BCL-2, CIAP-2, CIAP-1, C-FLIP* and *A1*; and pro-apoptotic *NOXA*, *FAS*, and *BAX* that control apoptosis^(14)^. Tognon et al. reported the deregulation of the expression of molecules of the extrinsic route, such as *FAS*, *FASL*, *FAIM*, *C-FLIP*, and the receptors of TRAIL-DR4 and DR5 in PV, ET, and MF^(16)^, as well as of the intrinsic route such as increased expression of anti-apoptotic genes *A1*, BCL-2, *BCL-X*_L_, and *BCL-W*, and decreased expression of genes *BID* and *BIM*_EL_ in leukocytes in ET and MF^(15)^. Additionally, the resistance of cells of patients to apoptosis in functional trials with classic apoptogenic inducers was described^(16)^, besides the association of the percentage of mutated alleles JAK2V617F with the expression of the pro-apoptotic genes *BAX*, *BIK*, and *BAD*, as well as the correlation between the expression of genes *A1*, *BAX*, and *BIK* and the *PRV1* gene, a cellular surface receptor involved in cellular proliferation processes^(15)^. The alterations of expression of molecules involved in the regulation of apoptosisin MPNs already described in literature are compiled in figure 1.

All these studies described above show modifications in the expression of molecules that participate in the regulation of intrinsic and extrinsic routes of apoptosis, as well as the findings of functional studies that showed resistance to apoptosis, indicating that the deregulation of apoptosis in MPNs is a mechanism involved in the pathophysiology of these diseases. The findings of association between the alterations and the presence of the JAK2V617F mutation in patients allowed us to infer that the elevated tyrosine kinase activity interferes in the expression of various genes related to cell proliferation and death. The relation between the expression of apoptosis molecules with JAK2 or STAT5 has been shown in literature for a long time. In models of MPNs, Gozgit et al. showed the effect of JAK2 inhibitors in the expression of *BCL-X*_L_ and *BAD* ^(24)^, and Rubert et al. demonstrated the interrelation between the activity of JAK2 with the expression of *BIM*, *MCL-1*, and *BCL-X*_L_^(25)^.

Understanding of the pathophysiological mechanisms is necessary for the development of treatments with specific targets. Identification of the main molecules that are altered in MPNs allows the development of drugs more directly targeted to the pathophysiology of the disease, with high efficacy, fewer adverse effects, contributing to compliance of the patients with treatments.

### Treatment of chronic myeloproliferative neoplasms

Currently, patients with MPNs are treated with cytoreduction (hydroxycarbamide), immunomodulators (e.g., oral interferon alpha – IFN-α – and thalidomide), cytokine blocking agents (antagonists of interleukin – IL –5 and 6), and inhibitors of JAK2 tyrosine kinase^(26)^.

Since the description of the JAK2V617F mutation, in 2005, the search has begun for a drug that had this tyrosine kinase as target and could be used in the treatment of patients with MPNs. In 2011, there were some JAK kinase inhibitors such as INCB018424 (ruxolitinib), TG101348, SB1518, and CEP-701 (Lestaurtinib)^(27)^ in an advanced phase of a clinical trial. Some studies with these drugs showed that all of them reduced splenomegaly and constitutional symptoms, without altering molecular or histological characteristics present in the MPNs^(27)^. In November 2011, INCB018424 (Incyte/NOVARTIS) was approved by the Food and Drug Administration (FDA) for the treatment of patients with an intermediate and high risk MF (commercial name: Jakafi™, Incyte Corp.), after two phase II studies showed the reduction of splenomegaly and improvement of symptoms. These studies also showed that both positive and negative patients responded to treatment with this inhibitor. Recent evidence has shown that the treatment could change the course of the disease, but studies have not shown significant changes in the degree of fibrosis and in the reduction of allele burden for patients with JAK2V617F^(28,29)^.

One relevant aspect in the development of new therapies for neoplasms would be the possibility of designing new drugs that target molecules fundamental for the regulation of cell signaling routes involved in cell proliferation and death, such as the apoptosis route. Inhibition of the BCL-2 family anti-apoptotic molecules, with molecules that mimic the BH3-only domain (ABT-737, AT-101, among others), with antisense oligonucleotides or interference RNA, are approaches investigated in the treatment of neoplasms^(30)^. This strategy that intends to sensitize cells to apoptosis seems promising for MPNs, since alterations in the expression of apoptosis regulating molecules were described in these disorders. IAPs antagonist molecules and antisense oligonucleotides for IAPs are investigated as therapies for neoplasms, for example, of the lungs, with drugs in pre-clinical and clinical phases of testing^(2)^.

For the future, it is conceivable to imagine the possibility of using molecules involved in apoptosis as prognostic and diagnostic markers for these diseases.
